# EfABI4 Transcription Factor Is Involved in the Regulation of Starch Biosynthesis in *Euryale ferox* Salisb Seeds

**DOI:** 10.3390/ijms23147598

**Published:** 2022-07-08

**Authors:** Peng Wu, Yue Zhu, Ailian Liu, Yuhao Wang, Shuping Zhao, Kai Feng, Liangjun Li

**Affiliations:** 1 School of Horticulture and Plant Protection, Yangzhou University, Wenhui East Road No. 48, Yangzhou 225009, China; wupeng@yzu.edu.cn (P.W.); zy19970814@163.com (Y.Z.); liuailianyz@163.com (A.L.); wangyuhaoyzu@163.com (Y.W.); zhaoshuping@yzu.edu.cn (S.Z.); fengkai@yzu.edu.cn (K.F.); 2 Joint International Research Laboratory of Agriculture and Agri-Product Safety of Ministry of Education of China, Yangzhou University, Yangzhou 225000, China

**Keywords:** ABA, starch, *E. ferox* (*Euryale ferox* Salisb.), transcriptome, EfABI4 transcription factor, *EfSS1*

## Abstract

Starch is the final product of photosynthesis and the main storage form in plants. Studies have shown that there is a close synergistic regulatory relationship between ABA signal transduction and starch biosynthesis. In this study, we employed RNA sequencing (RNA-Seq) to investigate transcriptomic changes of the *Euryale ferox* seeds treated by exogenous ABA. The differentially expressed genes engaged in the “Starch and sucrose” and “TCA cycle” pathway. Furthermore, the key transcription factor EfABI4 in ABA signaling pathway and the key genes of starch biosynthesis (*EfDBE1*, *EfSBE2*, *EfSS1*, *EfSS2*, *EfSS3*, *EfSS4* and *EfGBSS1*) were significantly up-regulated. Further, the *Euryale ferox* plant was treated with ABA, it was found that the total starch content of *Euryale ferox* seeds at different development stages was significantly higher than that of the control, and the key genes of starch synthesis in *Euryale ferox* seeds were also significantly up-regulated. Finally, yeast one-hybrid and dual luciferase assay proved that EfABI4 can promote the expression of *EfSS1* by directly binding to its promoter. Subcellular localization results showed that EfABI4 protein was located at the nucleus and EfSS1 protein was located in the cytomembrane. These findings revealed that ABA promotes starch synthesis and accumulation by mediating EfABI4 to directly promote *EfSS1* gene expression, which is helpful for understanding starch synthesis in seeds.

## 1. Introduction

ABA (abscisic acid) is one of the five major plant hormones, and plays an important role in regulating plant development [1]. ABA is closely related to the synthesis and transportation of starch and carbohydrate metabolism in plants. It affects the transport of assimilates by promoting the breakdown, absorption and unloading of sucrose from plant tissues and ultimately promotes starch formation in the sink tissues [2,3,4,5,6]. During the growth and development of gladiolus and tomato fruits, glucose and fructose contents were positively correlated with endogenous ABA content [7,8]. In addition, there was a positive correlation between ABA content and starch accumulation rate in seeds of wheat and barley at the grain filling stage [9]. Furthermore, exogenous ABA treatment can increase the starch content of banana [10], lotus [11], blueberry and peach [12,13]. However, the mechanism of ABA regulation of starch synthesis is unclear.

Starch is the main carbohydrate formed by carbon fixation during photosynthesis and plays an important physiological role in the whole growth and development of plants [14]. Starch is also a major component of human food and is widely used in wine, paper, adhesives, textiles and biodegradable plastics [15]. Starch is divided into two types: amylose and amylopectin. Amylose is linear and has no branching spiral structure; it is composed of glucose units connected by α-1,4-glycosidic bonds. Amylopectin is a highly branched glucose polymer, consisting of 24 to 30 glucose residues connected end to end by α-1,4-glycosidic bonds, with α-1,6-glycosidic bonds at the branch chain. It is the main component of starch [16]. Starch biosynthesis is regulated by different hormonal signals, such as abscisic acid (ABA), gibberellin (GA) and indole-3-acetic acid (IAA) [17,18,19,20,21]. The biosynthesis of starch involves various enzymes, such as ADPG-pyrophosphorylase (AGPase), starch synthase (SS), starch synthase (GBSS), starch branching enzyme (SBE) and starch debranching enzyme (DBE) [22,23,24,25]. Many studies have shown that starch metabolism is closely related to ABA in plants. In cassava, the rate of starch synthesis and accumulation increased with the increase in ABA content [26]. In tomato, glucose and fructose contents in peel, parenchyma and glial tissue were positively correlated with endogenous ABA content [8]. In rice, ABA content in grains of large-grain rice varieties was higher than that of small-grain rice varieties [27].

Abscisic acid insensitive 4 (ABI4) is a key transcription factor in ABA signaling. *ABI4* is mainly involved in regulating glucose metabolism and thus affecting starch synthesis [28,29,30,31]. In *Arabidopsis*, the *abi4* mutant has a glucose and fructose insensitive phenotype [32]. ABI4 has also been shown to promote the synthesis of triacylglycerol (TAG) [33]. *ABI4* regulates the expression of starch synthesis-related genes *APL3* and *SBE2*, and starch degradation genes *SEX1* and *BMY8/BAM3*, thus affecting the synthesis and accumulation of starch [34]. In addition, *NnABI4* can activate and up-regulate the expression of *NnSS1*, which promotes starch synthesis in *Nelumbo nucifera* [11]. Moreover, in maize endosperm, overexpressed *ZmABI4* can also up-regulate the expression of *AGPL2*, *AGPLS4*, *Sh2*, *SS1*, *GBSS1*, *SBE2a*, *SBE2b* and *PHOL* genes encoding AGPase, thus increasing starch content in maize endosperm [35].

*Euryale ferox* Salisb. (Nymphaeaceae) is an important characteristic aquatic vegetable in China, with high economic value and medical value [36,37]. It is very healthy food, abundant in nutritional and bioactive compounds such as carbohydrates, protein, flavonoids, vitamins, minerals and polyphenols. It has high medicinal value, such as reducing blood sugar, regulating blood fat, and anti-diabetic, anti-hyperlipidemic, and hepatoprotective effects. Starch is the main storage substance of *E. ferox* seeds [38]. In several local varieties of *Euryale ferox*, the starch content in seeds increases with the maturation of seeds, but the amylopectin content and the ratio of amylose to amylopectin are significantly different among varieties [39,40]. The content, composition and structure of *Euryale ferox* starch are closely related to the quality of *E. ferox* seeds [41,42]. At present, *EfSBE1* expression has been proven to be related to the difference in amylopectin in different varieties of *E. ferox* seeds, and *EfSBE3* can regulate the synthesis of amylopectin [41,43]. However, related studies on other types of starch synthase in *Euryale ferox* are still scarce. Therefore, studying how the starch synthesis of *E. ferox* seeds forms is important. In this study, the regulation mechanism of ABA on starch biosynthesis of *E. ferox* seeds was investigated.

To explore the physiological, biochemical, and molecular mechanisms underlying ABA regulated starch and to clarify the influence of ABA on quality of starch from *Euryale ferox*. We screened candidates for key differentially expressed genes after ABA treatment of *E. ferox* seeds by transcriptome. We further proved the effect of ABA on starch of *Euryale ferox* by treating the growth and development process of *Euryale ferox* with exogenous ABA. Finally, we demonstrated the molecular regulatory mechanism using Y1H assays, subcellular localization and dual luciferase assay. This study provides a solid theoretical basis for ABA regulation of *E. ferox* seed starch synthesis.

## 2. Results

### 2.1. Transcriptome Analysis of ABA Treated on Euryale ferox Seeds

The Illumina Novaseq 6000 system was used to deeply sequence the total RNA of six samples (ABA treated and control). In total, 264,860,112 high-quality reads were obtained by sequencing, and 39.74 Gb of clean data were obtained by further quality screening and detection (Figure S1). By comparing the raw counts of the ABA-treated and control groups, 5102 genes with significant differential expression (*p* < 0.05 and Log2FC ≥ 1) were obtained. Among them, 2745 genes were up-regulated and 2345 genes were down-regulated in the ABA treatment group, indicating that more genes responded positively to ABA treatment (Figure S2).

**Figure 1:**
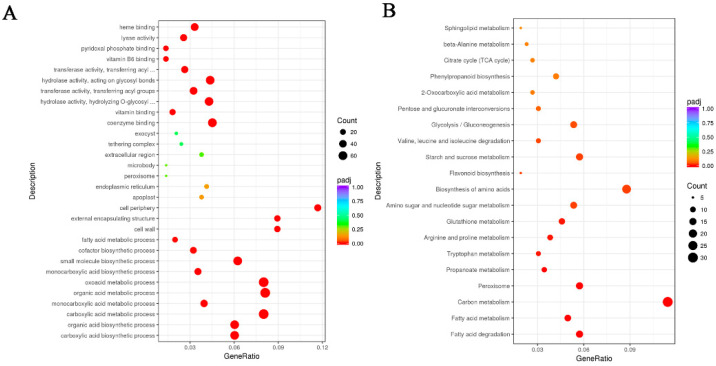
(**A**) GO enrichment of differentially expressed genes in *Euryale ferox* Salisb. (**B**) KEGG enrichment of differentially expressed genes in *Euryale ferox* Salisb.

In order to study the specific effects of exogenous ABA treatment on *E. ferox* seed, differentially expressed genes (DEGs) were classified by GO function. “Vitamin binding protein” and “pyruvate kinase activity” were enriched in “cell component”. “Carboxylic acid biosynthesis”, “organic acid biosynthesis” and “fatty acid metabolism” were enriched in “biological process”. “Catalytic activity” and “binding” were enriched in “molecular function” (Figure 1A). These results suggest that the differentially expressed genes may be involved in the starch biosynthesis process of *E. ferox* seeds. The KEGG analysis found that the most of the up-regulated DEGs were significantly enriched in “Starch and sucrose metabolism”, “Carbon metabolism”, “TCA cycle”, “Biosynthesis of amino acids”, “Phenylpropanoid biosynthesis” and “Galactose metabolism” (Figure 1B). The results of KEGG enrichment analysis were consistent with those of GO functional enrichment.

### 2.2. DEGs Related to Starch Biosynthesis

**Figure 2:**
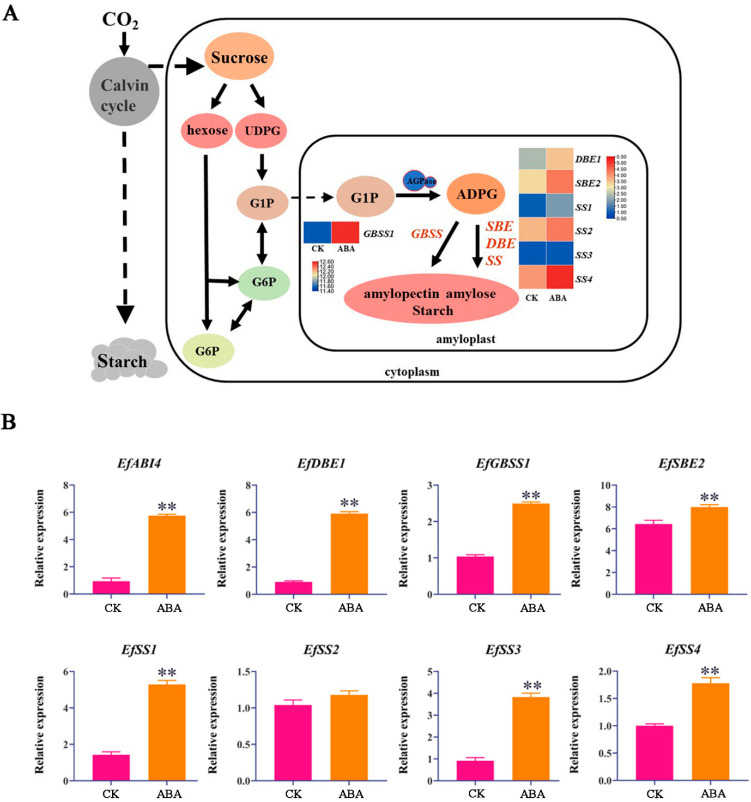
(**A**): Differentially expressed gene analysis of the starch biosynthesis pathway in response to ABA. (**B**): The relative expression levels of *EfABI4* and starch biosynthesis-related genes after ABA treatment. The ‘**’ above the histogram indicated the statistical significance at the level of 0.01 (*p* < 0.01).

According to GO and KEGG enrichment analysis, the large number of markedly regulated DEGs were significantly enriched in starch and sucrose metabolic pathways. The four key enzymes of starch synthesis are GBSS, SBE, SS and DBE. Among them, *EfGBSS1*, *EfDBE1*, *EfSBE2*, *EfSS1*, *EfSS2* and *EfSS4* were up-regulated after ABA treatment (Figure 2A). Further exploration of differentially expressed genes in the starch synthesis pathway is helpful to study the ABA regulatory mechanism of starch biosynthesis. At the same time, we found that the relative expression level of *EfABI4*, a key ABA response gene, was significantly higher than that of the control, indicating that the expression of *EfABI4* gene in *E. ferox* seeds was induced by exogenous ABA.

In order to verify the accuracy of RNA-seq results, the relative expression levels of *EfDBE1*, *EfGBSS1*, *EfSBE2*, *EfSS1*, *EfSS3*, *EfSS4*, and *EfABI4* were significantly up-regulated by qRT-PCR (Figure 2B and Table S2). These results were consistent with the expression level of differential genes in transcriptome results, indicating that the transcriptome analysis results were reliable.

### 2.3. ABA Treatment on Starch Biosynthesis during Euryale ferox Salisb Development

**Figure 3:**
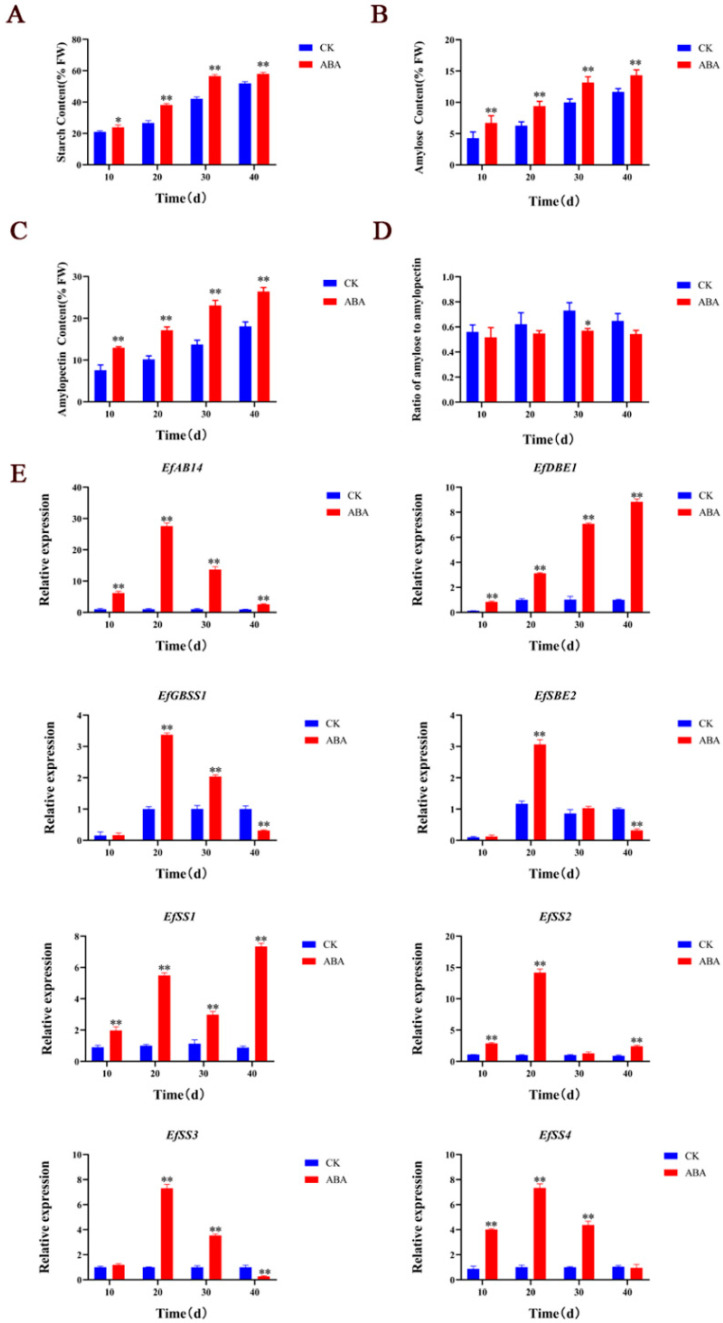
(**A**): The effects of ABA on total starch content in *E. ferox* seeds. (**B**): Effects of ABA on amylose synthesis in *E. ferox* seeds. (**C**): Effects of ABA on amylopectin synthesis in *E. ferox* seeds. (**D**): Effect of ABA on amylose to amylopectin ratio in *E. ferox* seeds. (**E**): The relative expression levels of *EfABI4, EfDBE1, EfGBSS1, EfSBE2, EfSS1, EfSS2, EfSS3* *and EfSS4* after ABA treatment during *Euryale ferox* Salisb development. The ‘*’ or ‘**’ above the histogram indicated the statistical significance at the level of 0.05 or 0.01 (*p* < 0.05; *p* < 0.01).

To further verify the transcriptome sequencing results, exogenous ABA was applied to the leaves of *Euryale ferox* during growth and development, and then the total starch content and the expression levels of key genes for starch synthesis were measured in *E. ferox* seeds at different development stages. In this experiment, the principle of triple biological repetition was strictly followed. Meanwhile, the same ecological environment conditions were maintained between control plants and ABA-treated plants, and the experimental arrangement was reasonable and rigorous. After exogenous ABA treatment, the contents of total starch, amylose, and amylopectin in *E. ferox* seeds at different developmental stages were higher than those in the control (Figure 3A–C and Tables S3–S5), especially at 20–40 days after flowering (Figure 3D and Table S6). In addition, the ratio of amylose to amylopectin decreased gradually and significantly at 30 days after flowering. Then, qPCR showed that after ABA treatment, the expression levels of *EfABI4* gene were higher than CK at different developmental stages, and the relative expression levels of starch synthesis-related genes *EfDBE1*, *EfGBSS1*, *EfSBE2*, *EfSS1*, *EfSS2*, *EfSS3* and *EfSS4* were also higher than CK, especially at 20 days after flowering (Figure 3E, Table S7). These results are consistent with the transcriptome results. Whether ABA mediates starch synthesis in *E. ferox* seeds by *EfAB14* remains to be studied.

### 2.4. EfAB14 Directly Promotes the Expression of EfSS1

**Figure 4:**
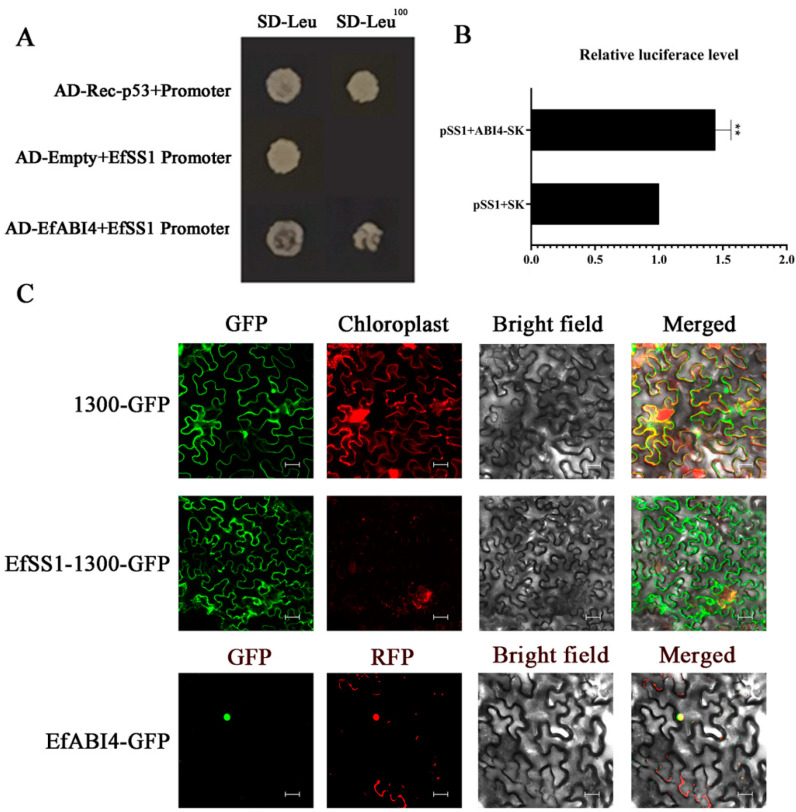
(**A**): Binding activities of EfABI4 protein with the promoters of *EfSS1* detected yeast one-hybrid assays. (**B**): The relative FLUC/RLUC activities of *EfSS1* promoters activated and repressed by EfABI4 in tobacco protoplasts. The relative FLUC/RLUC activity of the negative control was set at 1. ** indicates a significant difference at *p* < 0.01. (**C**): Subcellular localization of EfSS1 and EfABI4 (EfSS1 and EfABI4 were inserted into the pCAMBIA1300-sGFP vector). Scale bars, 10 μm.

To verify whether *EfABI4* can directly regulate the expression of *EfSS1*, *EfSS4* and *EfGBSS1*, we conducted a yeast one-hybrid experiment. As shown in Figure 4A, EfABI4 can bind to the promoter of *EfSS1*, indicating that *EfABI4* can directly regulate the expression of *EfSS1*. The regulation of *EfAB14* on *EfSS1* was studied by double luciferase method. The results showed that the co-expression of *EfABI4* and *EfSS1* increased the relative lucdiferin level in tobacco leaves by 1.44 times (Figure 4B and Table S8). Y1H and dual luciferase assay confirmed that EfAB14 could bind the *EfSS1* promoter and promote the expression of *EfSS1* in *E. ferox* seeds. In addition, subcellular localization results indicated that EfABI4 was located at the nucleus, and EfSS1 was located in the cytomembrane (Figure 4C).

## 3. Discussion

As an important plant hormone, ABA regulates many physiological processes of plant growth and development [44,45,46]. Previous studies have shown that ABA regulates sucrose transport and metabolism by inducing transcription of related genes through signal transduction into the nucleus [47]. ABA injections into strawberries could promote the content of soluble sugar in fruit [46]. ABA can promote the long-distance transport of sugar by enhancing phloem area and promoting the expression of some hexose transporter genes [48]. Moreover, treatment of wheat and barley ears with low concentration of ABA can promote assimilate transfer to grains [49]. Therefore, transcriptome sequencing was performed on *E. ferox* seeds after ABA treatment and we found that starch and sugar metabolic pathways were enriched. The *Euryale ferox* plants were treated with ABA during development, and total starch, amylose, amylopectin and expression of related genes in *E. ferox* seeds also increased significantly. These results were consistent with previous research results, but the specific regulation mechanism needs to be further explored.

**Figure 5:**
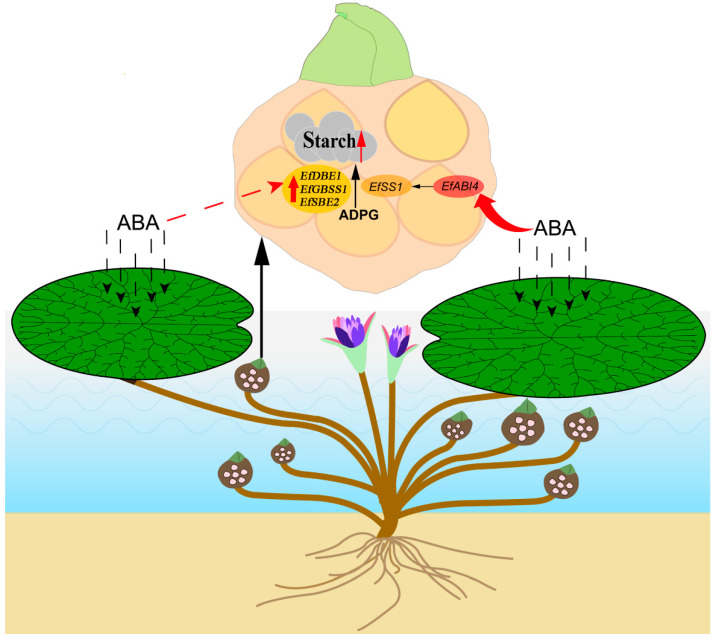
The model of ABA regulating starch biosynthesis in *E. ferox* seeds.

ABI4, as a key transcription factor in ABA signaling, is associated with starch synthesis in plants [50,51]. In previous studies, AtABI4 in *Arabidopsis* directly binds to CE1-like elements in the promoter regions of *SBE2.2* and *APL3* genes, up-regulating the expression of *SBE2.2* and *APL3*, thereby increasing starch content in seeds [34]. In rice, the expression of *OsAPL3* gene is regulated by both sucrose and ABA levels [52]. *ZmABI4* can up-regulate the expression of *ZmSS1* and *ZmGBSS1* genes and increase the starch content in maize endosperm [35]. In addition, it was recently reported that NnABI4 in *Nelumbo nucifera* can directly bind to the promoter of *NnSS1* and up-regulate its expression, ultimately promoting starch synthesis in rhizomes [11]. AtABI4 drives the expression of downstream genes by binding to the CE1-like motif element in the promoter region of the sugar response gene, such as the large subunit ApL3 of ADP-glucose pyrophosphorylase and the starch branch enzyme SBE2.2 in the starch synthesis pathway [53]. In our study, the promoter of *EfSS1* in *E. ferox* seeds contains CACCG, a coupling element of CE1. Y1H assay and dual luciferase assay confirmed that EfABI4 could bind to the *EfSS1* promoter and promote the expression of *EfSS1* (Figure S3). These results prove that *EfAB14* plays a key role in regulating the biosynthesis of starch from *E. ferox* seeds. Therefore, an important scientific issue is to explore the specific regulatory mechanism of *ABI4* on the anabolism of starch and sugar, and further research is needed. At the same time, the expression levels of *EfDBE1*, *EfGBSS1*, *EfSBE2*, *EfSS1*, *EfSS2*, *EfSS3* and *EfSS4* were also significantly up-regulated after ABA treatment, which was consistent with the increasing trend of starch content in *E. ferox* seeds. Therefore, the model of ABA mediating starch synthesis in *E. ferox* by *EfAB14* was constructed (Figure 5), but whether ABA also mediates starch synthesis in *E. ferox* by other ABA response factors needs to be further explored. 

## 4. Materials and Methods

### 4.1. Plant Materials and ABA Treatment

*Euryale ferox* ‘ZS_01’ was used as experimental material and planted in Yangzhou University Aquatic vegetable test base in summer 2021. *Euryale ferox* ‘ZS_01’ is grown in the open air under natural conditions (32°23′ N, 119°25′ E). *E. ferox* seeds were collected 25 days after flowering, soaked in 100 mg/L ABA (Sheng Gong, Shanghai, China) solution, and sampled after soaking for 0 h and 18 h. They were quickly washed and placed in liquid nitrogen and refrigerated at −80 ℃ for use, with three biological replicates for each sample.

In addition, after the *Euryale ferox* plants entered the reproductive growth stage, 50 mL 5 mg/L ABA solution was applied to the leaves once every 7 days for 3 times in total (12 August 2021, 19 August 2021, 26 August 2021). *E. ferox* seed samples were collected at 10, 20, 30 and 40 days after flowering. Three replicates were set for each treatment. The seeds were quickly frozen, made into powder, and stored at −80 ℃.

### 4.2. RNA Extraction and Sequencing

Total RNA was extracted from *E. ferox* seeds after ABA treatment using plant RNA extraction kit (Dalian Takara, Dalian, China). The RNA integrity of ABA treated seeds at 0 h and 18 h was analyzed by agarose gel. After passing the quality inspection process, a cDNA library was constructed according to the steps of the cDNA library construction kit. After the library was constructed, the library was checked and sequenced using Illumina Novaseq 6000 system after reaching the standard. The accuracy of subsequent analysis was positively correlated with the quality of reads, so raw reads obtained by sequencing needed to be detected. In order to ensure high data quality, redundant, complex and low-quality reads were screened and cut out, and the high-quality reads obtained were called clean data. The filtered sequenced sequence was compared with the reference genome *E. ferox*. Trinity software was used for a series of sequence assembly of high-quality sequencing data, and long and high-quality transcripts were obtained for subsequent research and analysis [54].

In order to obtain more comprehensive gene function information, we carried out rigorous bioinformatics analysis on the obtained *E. ferox* Unigene, including differential expression gene analysis and differential gene GO and KEGG enrichment analysis. After differential gene expression analysis, GO enrichment analysis was performed to further annotate the genes and gene products. Finally, Unigene obtained from *E. ferox* was compared with KEGG, a database with genome deciphering function, for the convenience of subsequent research and analysis.

### 4.3. Reverse Transcription-Quantitative PCR (RT-qPCR)

The extracted RNA was reverse transcribed into cDNA using HiScript^®^IIl RT SuperMixfor qPCR (Cat No.10911; Yeasen, Shanghai, China). Quantitative reverse transcription polymerase chain reaction (QRT-PCR) was used to analyze the key genes of the starch synthesis pathway in *E. ferox* seeds. The qRT-PCR reaction was 20 µL, including 10 µL 2 × ChamQ SYBR qPCR Master Mix (Vazyme Biotech, Co., Ltd.), 0.8 µL positive and negative primer mixture, 1.0 µL cDNA template and 8.2 µL ddH_2_O, respectively. Primer Premier 5.0 was used for Primer design. See Supplementary Table S1 for the gene specific primer sequences. β-actin (*EfUBQ5*) gene was used as an internal gene expression control: the gene was amplified with forward primer 5′-GTGAAGGCGAAGATCCAGGACAAG-3′ and reverse primer 5′-CCACGAAGGCGAAGCACAAGG-3′. Amplification was performed on the CFX-96 Real-time PCR system (Bio-Rad, Hercules, CA, USA) using the following real-time fluorescent quantitative PCR program: 95 ℃ for 30 s, 95 ℃ for 10 s and 60 ℃ for 30 s for a total of 40 cycles. The relative gene expression was calculated by 2^−∆∆CT^ [55]. Three replicates were performed for each amplification reaction.

The photosynthetic parameters of leaves were measured for six consecutive days on the second day after the first spray of exogenous ABA. The portable photosynthesis measurement system LI-6400XTP can simultaneously measure the leaf photosynthetic rate (Pn), stomatal conductance, and transpiration rate (Ts).

### 4.4. Determination of Starch Content in ABA Treated Leaves

Fresh *E. ferox* seeds were ground with a mortar. The total starch content of 3 mg sample, amylopectin content of 0.005 g sample and amylose content of 0.01 g sample were determined by visible spectrophotometry. A Solarbio Detection Kit (Beijing Solarbio Science & Technology Co., Ltd.) was used to determine total starch, amylose and amylopectin contents. Starch content (mg/g) = 1.351X/0.003 × 1.11, X = (ΔAdetermination + 0.0756)/4.9517, ΔA is ΔA determination = (A determination-A blank) − (A’ determination -A’ blank); Amylose content (mg/g) = 2 × ΔA determination/ΔA standard/0.001; Amylopectin content (mg/g) = X × 5/0.005, X = (ΔAdetermination + 0.0006)/0.7564. Each measurement was repeated three times.

### 4.5. Y1H Assays

The full-length CDS of *EfABI4* was inserted into pGADT7 (Shanghai Oebiotech, Shanghai, China) to generate the AD-*EfABI4* structure. The *EfSS1* promoter fragment was cloned into pAbAi (Shanghai Oebiotech, Shanghai, China) vector to generate pAbAi-*EfSS1*, which was linearized by BstBI (Dalian Takara, Dalian, China) and then transformed into Y1HGold yeast strain (Shanghai Oebiotech, Shanghai, China). The transformed cells were grown in the SD/-Ura plate for 3 days. After inhibiting the self-activation of the *EfSS1* promoter with Aureobasidin A (Dalian Takara, Dalian, China), the prey *ABI4* was transferred to the Y1HGold yeast (Shanghai Oebiotech, Shanghai, China) containing the *EfSS1* promoter. Subsequently, the yeast was spread on the SD/−Leu +AbA^100^ plate and allowed to stand and cultivate for 3–6 days.

### 4.6. Subcellular Localization

The encoding sequences of *EfABI4* and *EfSS1* were cloned into pCAMBIA1300-GFP by SacⅠ and XbaⅠ (Dalian Takara, Dalian, China) digestion sites (pSuper: GFP-*EfABI4*, GFP-*EfSS1*). The plasmid was transformed into *Agrobacterium tumefaciens* GV3101 and grown under appropriate antibiotics for subcellular localization analysis. *Agrobacterium*-mediated transient expression in 4-week-old *Nicotiana benthamiana* leaves was studied by syringe osmosis. After 72 h of dark growth, the GFP fluorescence of the samples was observed using a confocal laser scanning microscope (Zeiss, Jena, Germany). The primers are listed in Supplementary Table S1.

### 4.7. Transient Dual-Luciferase Detection

To further verify the binding activity of EfABI4 protein and *EfSS1* promoter, double luciferase assay was performed. Firstly, the recombinant plasmids *EfABI4*-pGreenII 62-SK and pro*EfSS1*-pGreenII 0800-LUC were transferred into *A. tumefaciens* GV3101 strain. Secondly, the bacteria were resuspended with the infection solution (100 mM Acetosyringone, 0.5 M MES (PH5.6) and 10 mM MgCl_2_) until the OD600 value was 1.0, and the bacterial solution containing *EfABI4*-pGreenII 62-SK and the bacterial solution pro*EfSS1*-pGreenII 0800-LUC were mixed in a ratio of 1:1, and then left to stand for 3 h. Then, the tobacco (*Nicotiana benthamiana*) was injected. According to the fluorescence value measured by the double reporting system, the fluorescence value of the target gene plasmid/the fluorescence value of the internal reference plasmid (i.e., F/R value) was calculated, and the ratio of the target gene plasmid to the control group and the standard error were calculated and the histogram was made.

## 5. Conclusions

In conclusion, we identified the expression profiles of a number of differentially expressed key genes, including *EfABI4*, *EfDBE1*, *EfGBSS1*, *EfSBE2*, *EfSS1*, *EfSS2*, *EfSS3* and *EfSS4*, which were highly correlated with the expression profiles of key starch biosynthesis-related genes. Moreover, we demonstrated that the EfABI4 transcription factor can directly bind to the *EfSS1* promoter, up-regulate the expression of *EfSS1* in *E. ferox* seeds, and promote the biosynthesis of starch in *E. ferox* seeds. This provides a practical basis for understanding the molecular regulatory network between ABA signal transduction and starch biosynthesis.

## Data Availability

Data are contained within the article or Supplementary Materials.

## Supplementary Materials

The following supporting information can be downloaded at: https://www.mdpi.com/article/10.3390/ijms23147598/s1.
