# Supplementary figures and images for: EfABI4 Transcription Factor Is Involved in the Regulation of Starch Biosynthesis in Euryale ferox Salisb Seeds
Source: Int J Mol Sci. 2022 Jul 8;23(14):7598. doi: 10.3390/ijms23147598 (PMC9317497; doi:10.3390/ijms23147598)

**A**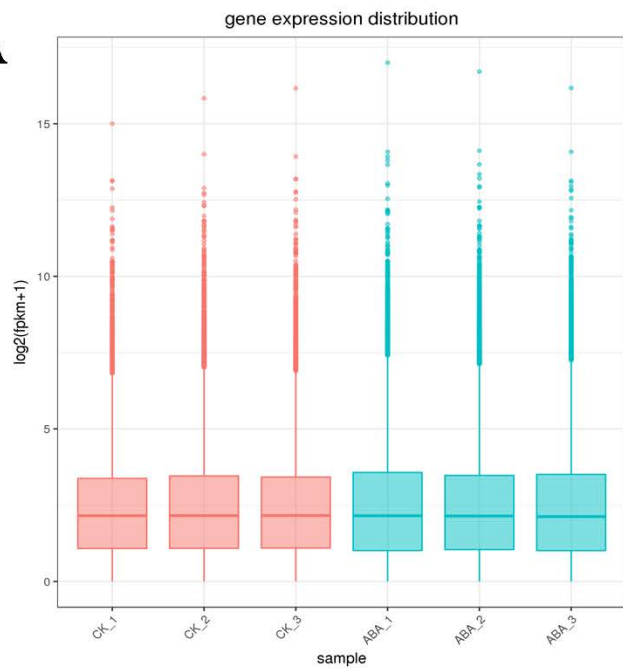**B**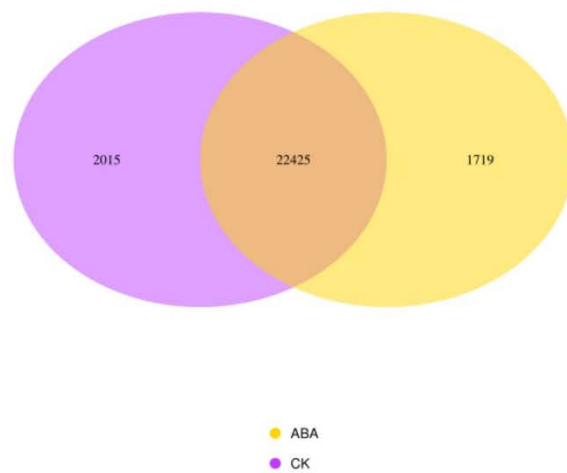**C**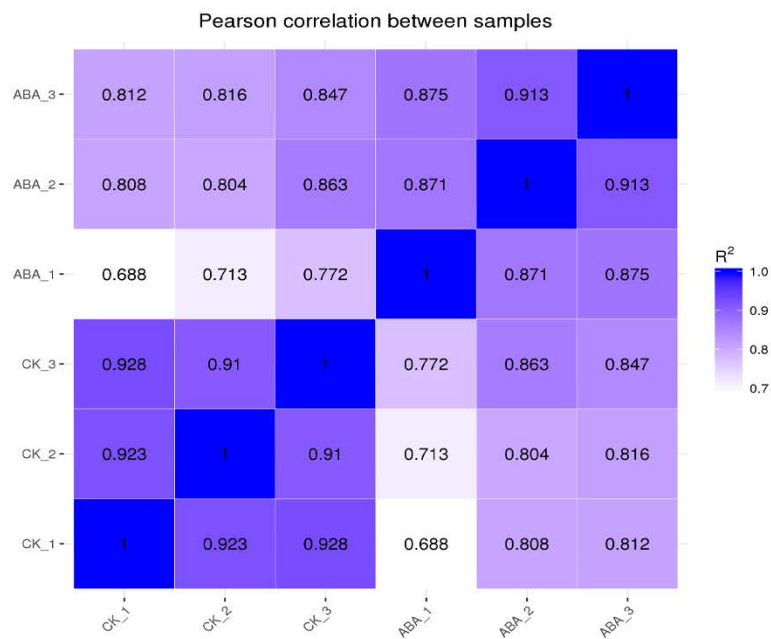

Supplement: Supplementary file 1 [file ijms-23-07598-s001.zip › Supplementary Figures/Fig.S1.pdf]

A

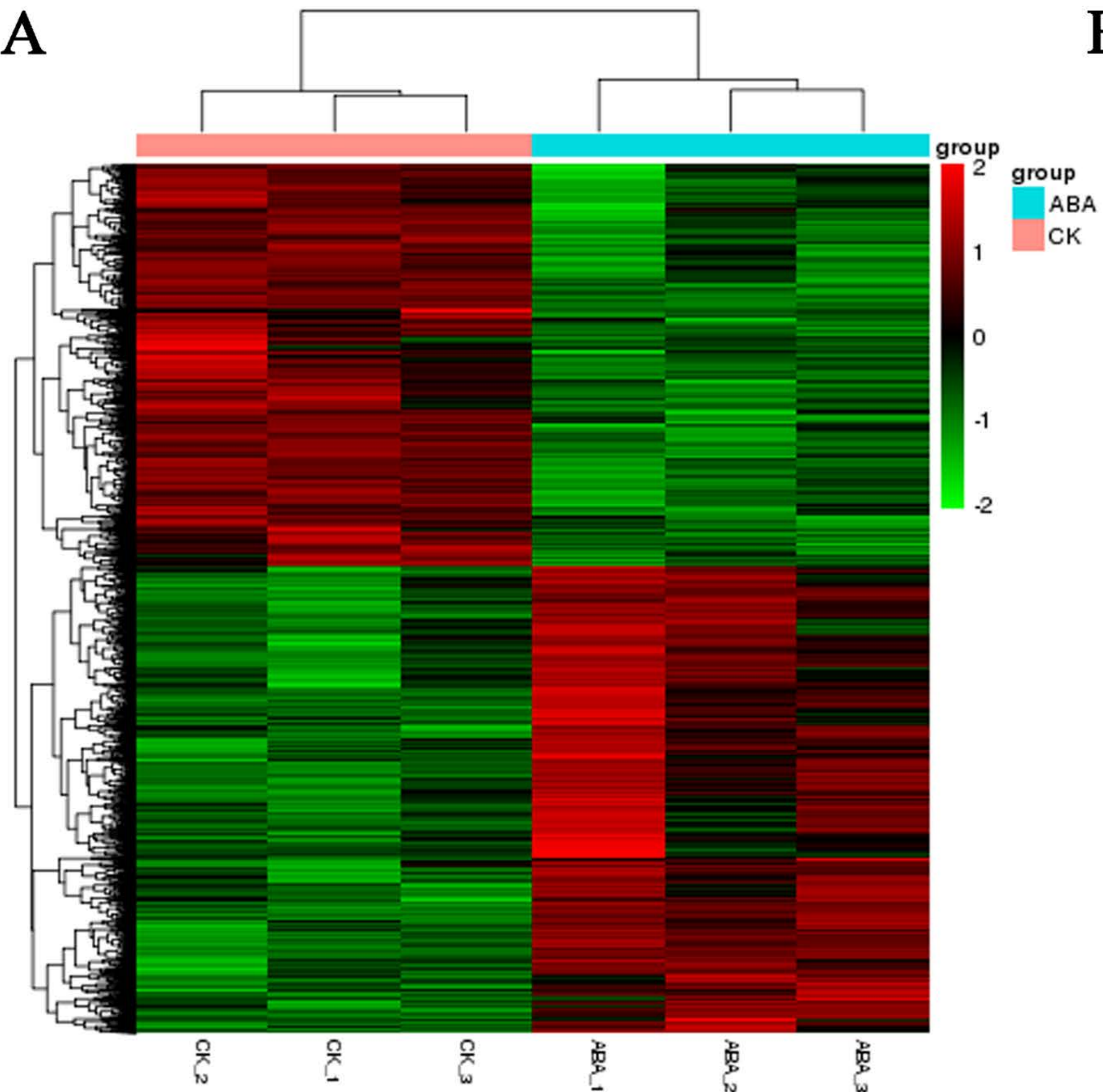

B

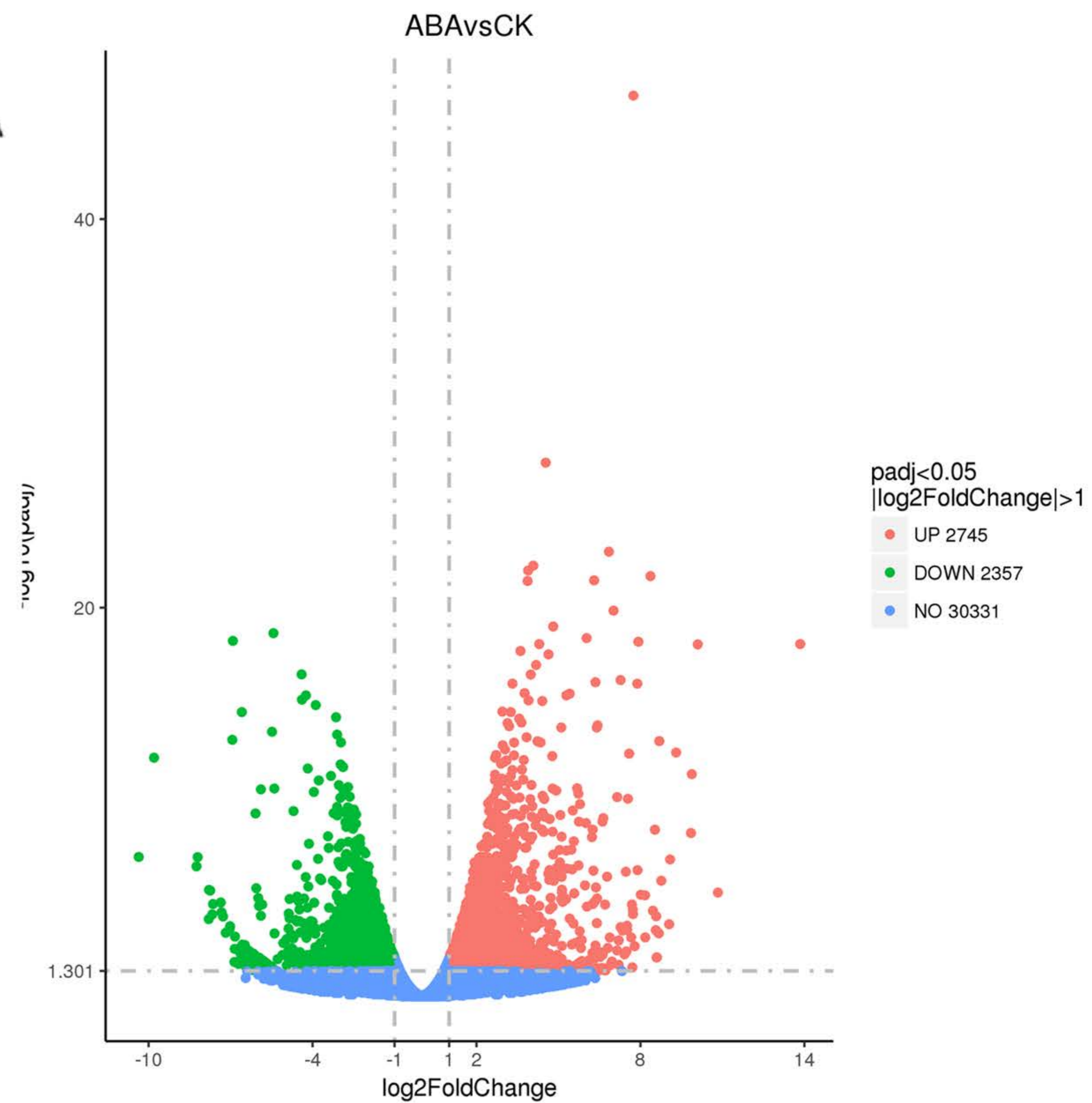

Supplement: Supplementary file 1 [file ijms-23-07598-s001.zip › Supplementary Figures/Fig.S2.pdf]

A

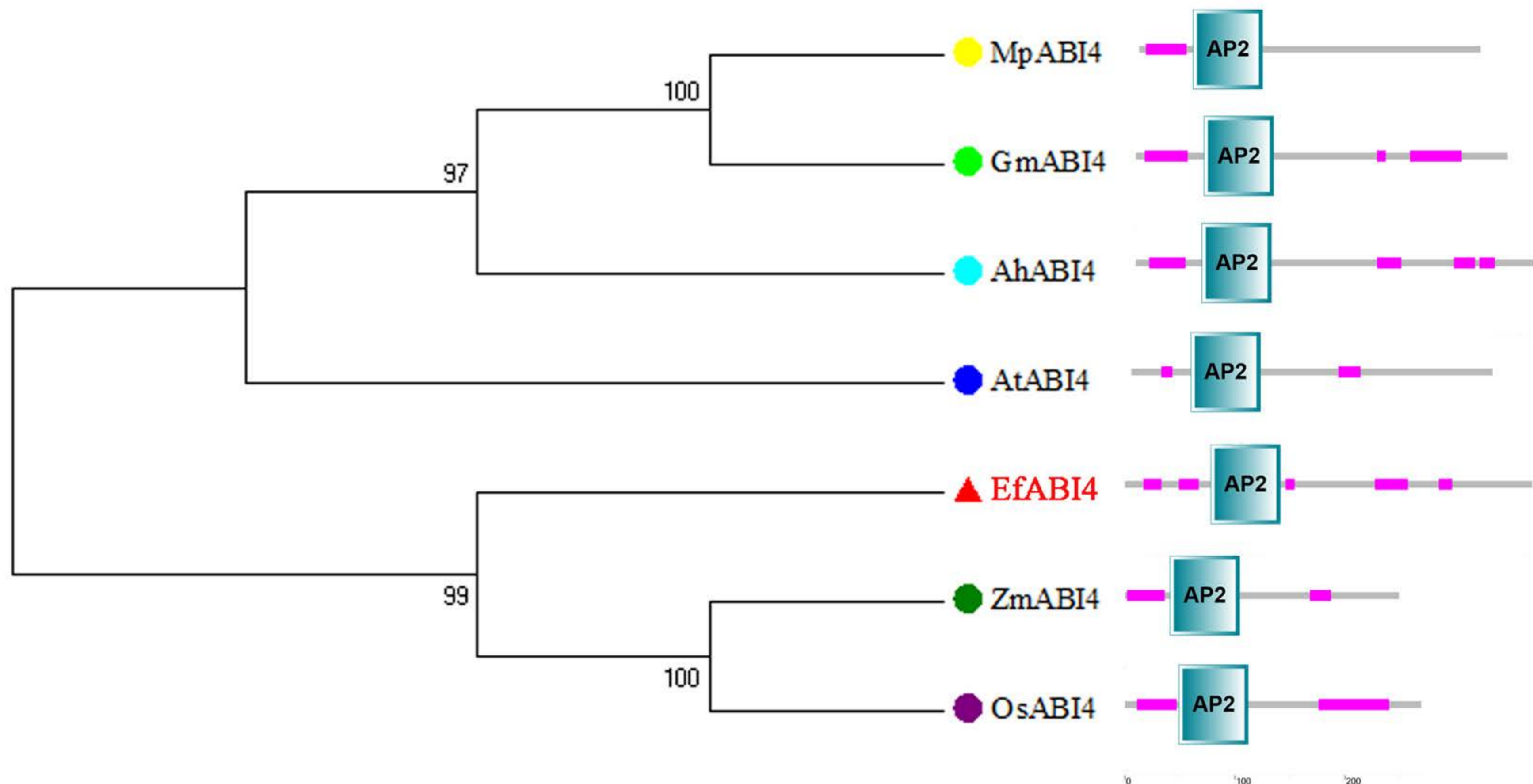

B

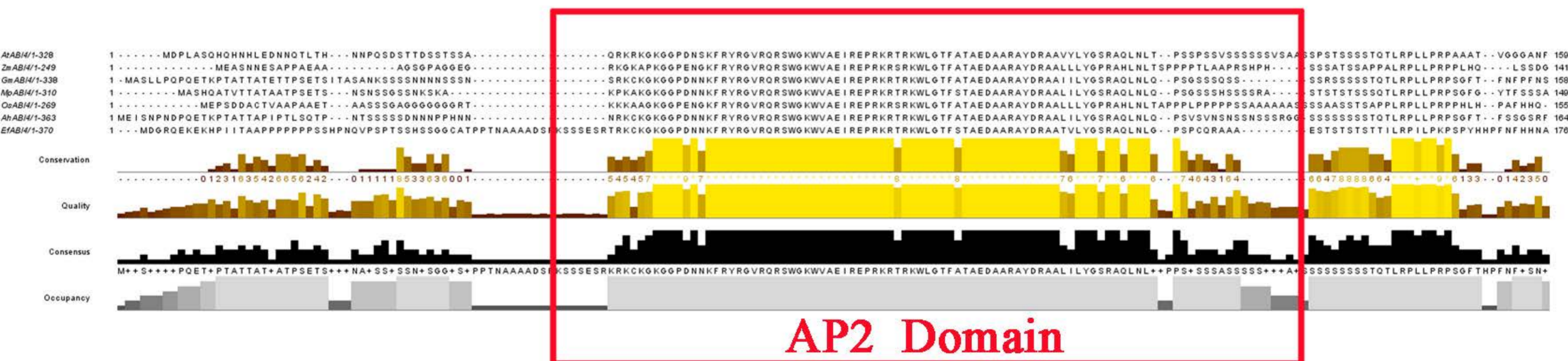

C

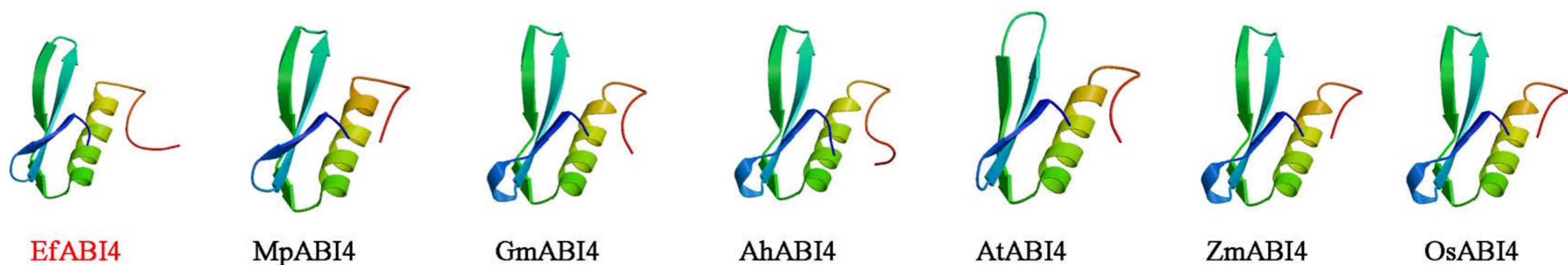

Supplement: Supplementary file 1 [file ijms-23-07598-s001.zip › Supplementary Figures/Fig.S3.pdf]
